# Spine-to-Dendrite Calcium Modeling Discloses Relevance for Precise Positioning of Ryanodine Receptor-Containing Spine Endoplasmic Reticulum

**DOI:** 10.1038/s41598-018-33343-9

**Published:** 2018-10-23

**Authors:** Markus Breit, Marcus Kessler, Martin Stepniewski, Andreas Vlachos, Gillian Queisser

**Affiliations:** 1 Goethe Center for Scientific Computing, Computational Neuroscience, Goethe University Frankfurt, Frankfurt, Germany; 2 Department of Neuroanatomy, Institute of Anatomy and Cell Biology, Faculty of Medicine, University of Freiburg, Freiburg, 79104 Germany; 3 Bernstein Center Freiburg, University of Freiburg, Freiburg, 79104 Germany; 4 Department of Mathematics, Temple University, Philadelphia, USA

## Abstract

The endoplasmic reticulum (ER) forms a complex endomembrane network that reaches into the cellular compartments of a neuron, including dendritic spines. Recent work discloses that the spine ER is a dynamic structure that enters and leaves spines. While evidence exists that ER Ca^2+^ release is involved in synaptic plasticity, the role of spine ER morphology remains unknown. Combining a new 3D spine generator with 3D Ca^2+^ modeling, we addressed the relevance of ER positioning on spine-to-dendrite Ca^2+^ signaling. Our simulations, which account for Ca^2+^ exchange on the plasma membrane and ER, show that spine ER needs to be present in distinct morphological conformations in order to overcome a barrier between the spine and dendritic shaft. We demonstrate that RyR-carrying spine ER promotes spine-to-dendrite Ca^2+^ signals in a position-dependent manner. Our simulations indicate that RyR-carrying ER can initiate time-delayed Ca^2+^ reverberation, depending on the precise position of the spine ER. Upon spine growth, structural reorganization of the ER restores spine-to-dendrite Ca^2+^ communication, while maintaining aspects of Ca^2+^ homeostasis in the spine head. Our work emphasizes the relevance of precise positioning of RyR-containing spine ER in regulating the strength and timing of spine Ca^2+^ signaling, which could play an important role in tuning spine-to-dendrite Ca^2+^ communication and homeostasis.

## Introduction

The endoplasmic reticulum (ER) is a multifunctional intracellular organelle, which consists of a complex three-dimensional network of connected endomembrane tubules, stacks and cisternae^1–4^. In neurons, the relevance of its strategic positioning is reflected by the fact that it reaches from the nucleus and soma into neurites, i.e., dendrites and axons, and it is frequently found in proximity of excitatory and inhibitory pre- and postsynaptic sides. This observation has coined the term “neuron within a neuron” for neuronal ER morphology^5^. While its role in synaptic protein synthesis, protein maturation, and transport is still debated, it is best-studied for its ability to release Ca^2+^ in a receptor-dependent manner, which modulates the capacity of synapses to undergo plastic changes^2,6–9^.

The ER consists of a complex, overlapping and partially cell- and region-specific Ca^2+^ handling machinery, including Ca^2+^ pumps and transporters^2^. In hippocampal neurons, for example, inositol trisphosphate receptors (IP_3_R) are present at high concentrations in dendritic shafts and cell bodies, whereas ryanodine receptors (RyR) are primarily found in dendritic spines and axons^10^ (see also^11^). In contrast, Purkinje cells of the cerebellum show high concentrations of IP_3_R also in dendritic spines^12,13^. Whether these receptors are evenly distributed along the spine ER compartment or rather clustered at strategic positions remains unknown. More recent work has also established a link between store-operated Ca^2+^ entry (SOCE), i.e., ORAI-STIM1-mediated Ca^2+^ signaling, and neuronal ER-mediated plasticity (e.g.^14,15^). Another major challenge in this field of research is the fact that the ER is a dynamic structure that can rapidly enter and leave pre-existing spines, while changing its position within individual ER-positive spines^16,17^. Hence, it is conceivable that spine-to-dendrite Ca^2+^ communication may critically depend on (1) whether or not a spine contains ER, (2) ER Ca^2+^ receptor composition, and (3) the precise ER morphology and position within a spine.

**Figure 1:**
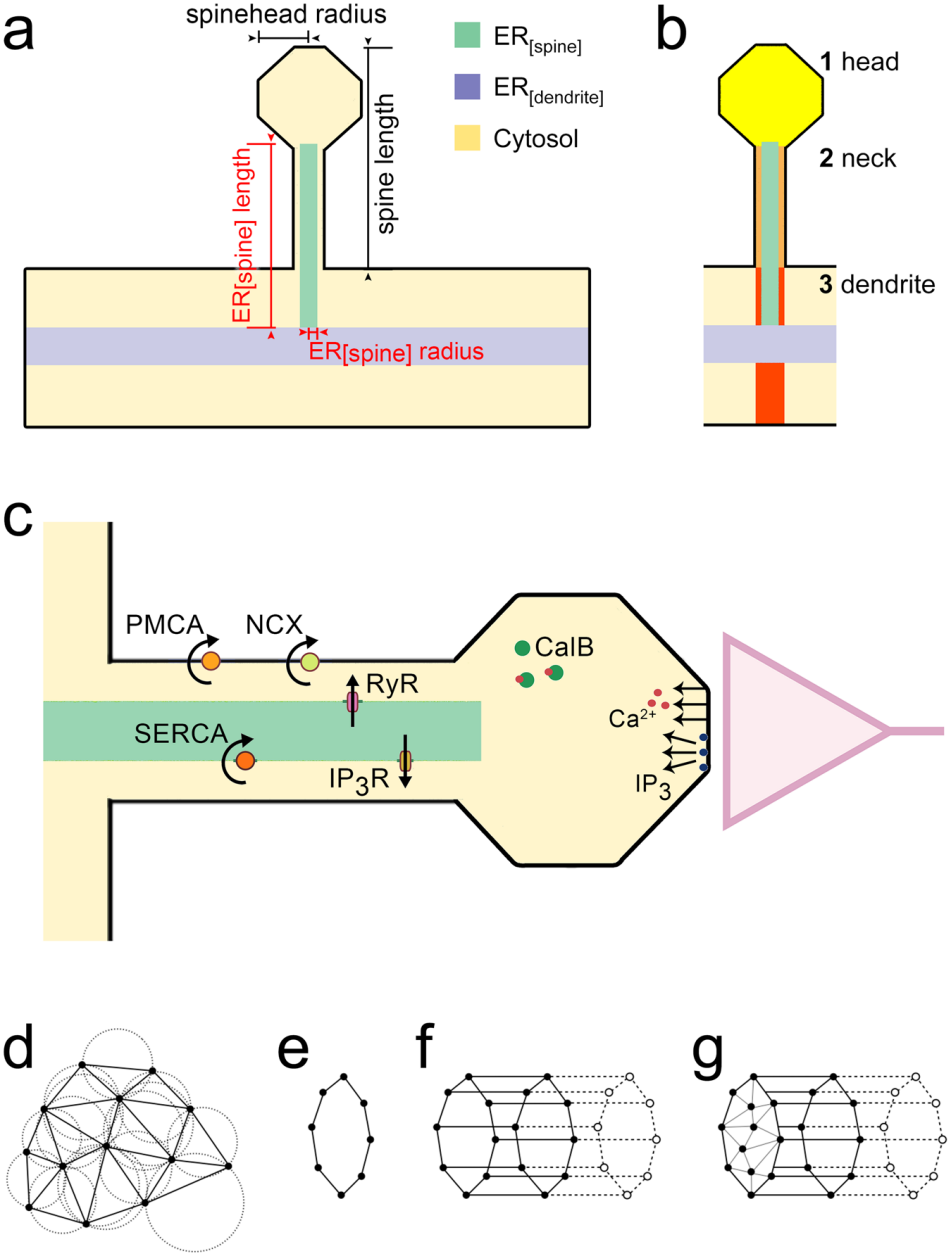
Spine Ca^2+^ modeling. (**a**) Schematic illustration of a single spine emerging from a dendrite containing endoplasmic reticulum (ER). Spine ER (ER_[spine]_, green) reaches into the spine compartment. The relevant parameters evaluated in this study are indicated. Spine morphology is based on average values of ^21^, who used stimulated emission depletion (STED) microscopy to determine parameters such as spine length, spine head size and spine neck width. **(b**,**c)** Upon release of Ca^2+^ ions and inositol trisphosphate (IP_3_) molecules in the head of the spine, changes in [Ca^2+^] are determined in the head (yellow), neck (orange) and dendritic region (red), respectively. The model accounts for Ca^2+^ exchange mechanisms on the plasma membrane (Na^+^/Ca^2+^ exchangers (NCX), plasma membrane Ca^2+^ -ATPases (PMCA)) and on the ER (inositol trisphosphate receptors (IP_3_R), ryanodine receptors (RyR), and sarco/endoplasmic reticulum Ca^2+^ -ATPases (SERCA)). **(d)** Sample 2D triangulation ${\mathscr{S}}=\{{T}_{1},\mathrm{...},{T}_{M}\}$ with fulfilled Delaunay condition, i.e., the unique circumcircle of each *T*_*i*_, which passes through the three triangular vertices, does not contain any vertices of the grid in its interior. **(e)** Piecewise linear approximation of a circle with 8 rim vertices as used for the construction of the dendrite, ER and spine structures in the spine generator. **(f)** Successive circle extrusion with creation of quadrilateral faces enclosing the emerging cylinder barrel during dendrite, ER and spine structure generation. **(g)** Exemplary triangulation of the planar hole at the left cylinder side as used for closing the encapsulated spine surface geometry. Further details are provided in Tables 1 and 2. See also supplemental movie.

In order to capture how distinct spine ER properties influence spine-to-dendrite Ca^2+^ communication, the three-dimensional intracellular architecture must be considered^18,19^. Therefore, we developed a new spine and ER generator for the simulation framework NeuroBox^20^ to parametrically design three-dimensional computational domains (Fig. 1a). Existing single-channel models of Na^+^/Ca^2+^ exchangers in the plasma membrane, as well as RyR, IP_3_R and sarco/endoplasmic reticulum Ca^2+^ ATPases (SERCA) on the ER membrane (see schematic in Fig. 1c) were adapted and integrated in a novel three-dimensional calcium model that is solved by established numerical methods (details provided in Methods). Using this novel framework, we systematically assessed the relevance of selected spine ER properties, i.e., length, width and presence of RyR and IP_3_R on spine-to-dendrite Ca^2+^ signaling.

## Results

### Passive spine ER has no major impact on spine-to-dendrite Ca^2+^ signaling

To assess the role of spine ER positioning in spine-to-dendrite Ca^2+^ communication, we first investigated Ca^2+^ signal propagation in a representative 3D spine model not containing any ER. The simplified morphology of the spine (Fig. 1a) is based on mean values obtained from stimulated emission depletion (STED) live cell microscopy experiments^21^. Ca^2+^ ions were released into the spine head with three distinct release profiles, i.e., a 1 ms release and two longer release periods with a 10 ms and 150 ms time constant, respectively. Changes in [Ca^2+^] were determined in the indicated regions of interest (Fig. 1b), i.e., in the spine head, neck and dendrite.

**Figure 2:**
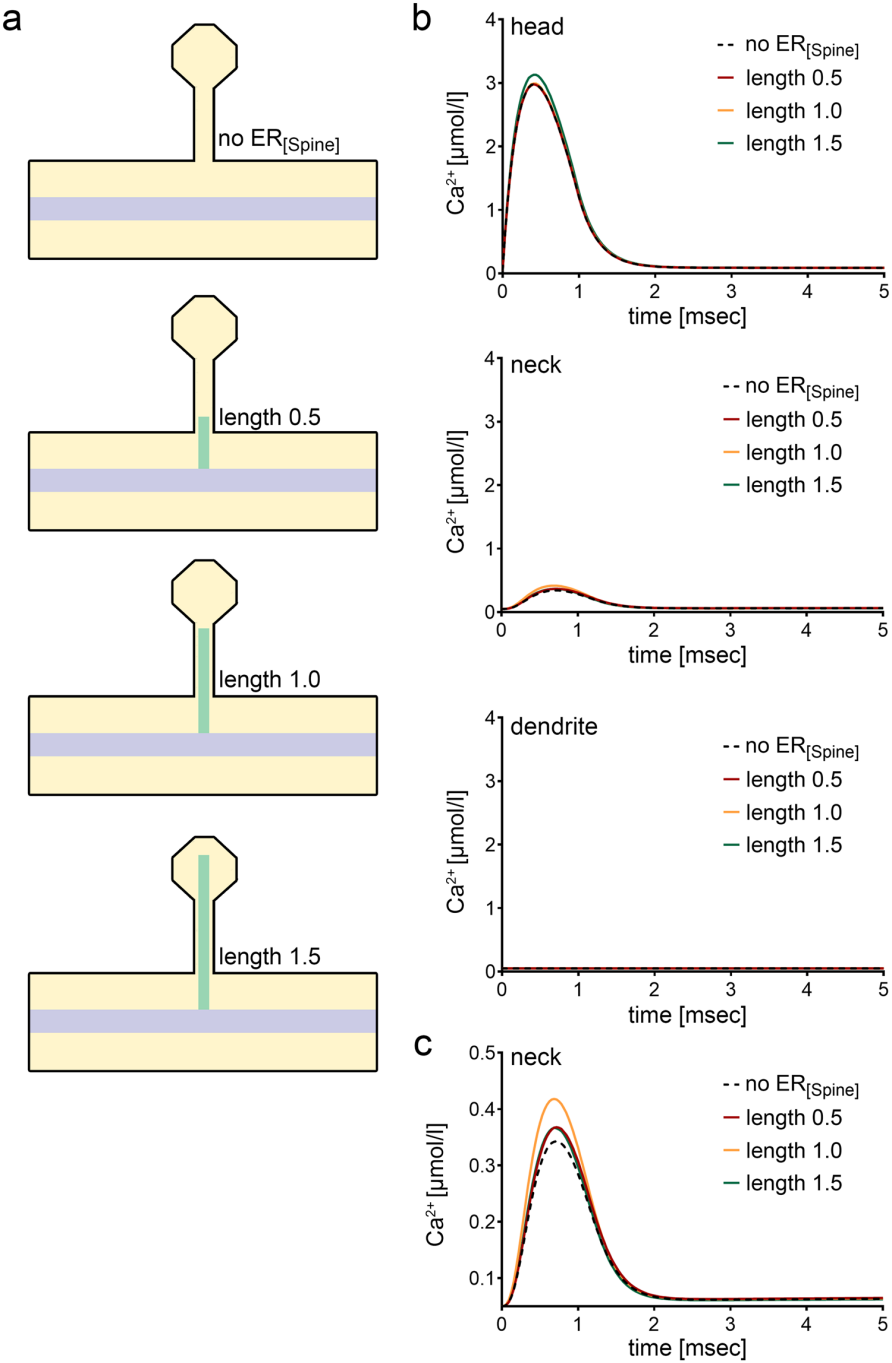
Effects of passive spine ER on spine-to-dendrite Ca^2+^ signaling. (**a**) Schematics of three selected morphological settings. Spine ER length is given in units of µm. **(b)** Ca^2+^ profiles for 1 ms initial Ca^2+^ release into the spine head (cf. Supplemental Fig. S1). Consistent with experimental data, spine-to-dendrite Ca^2+^ signaling does not occur in our simulations. Presence or length of passive spine ER has only minor effects on Ca^2+^ dynamics in the spine head and neck. Ca^2+^ buffering has a major impact in these simulations, thus inactivating spine-to-dendrite Ca^2+^ communication. **(c)** Minor effects of the passive spine ER on the Ca^2+^ profile are shown at higher magnification for the spine neck.

As shown in Fig. 2 for 1 ms initial Ca^2+^ release, only a small fraction of Ca^2+^ reaches the dendritic compartment (<1%; Fig. 2b). When the spine ER is passive, i.e., when it is present as a geometric obstacle, but without any Ca^2+^ exchange mechanisms that would allow Ca^2+^ exchange across the ER membrane, nearly identical and near-zero dendritic Ca^2+^ profiles for all ER lengths are observed (Fig. 2b)^22^. Similar results were obtained for the longer Ca^2+^ influx durations (c.f., Supplemental Fig. S1). From these results we conclude that spine-to-dendrite Ca^2+^ signaling is negligible in the no-spine-ER and the passive-spine-ER setting. Accordingly, precise positioning of a purely passive spine ER compartment has only minor effects on spine-to-dendrite Ca^2+^ signals in our experimental setting. This observation strengthens the case for active Ca^2+^ exchange across the spine ER membrane to enable spine-to-dendrite Ca^2+^ communication.

### Ryanodine receptor-containing ER promotes spine-to-dendrite Ca^2+^ signaling

**Figure 3:**
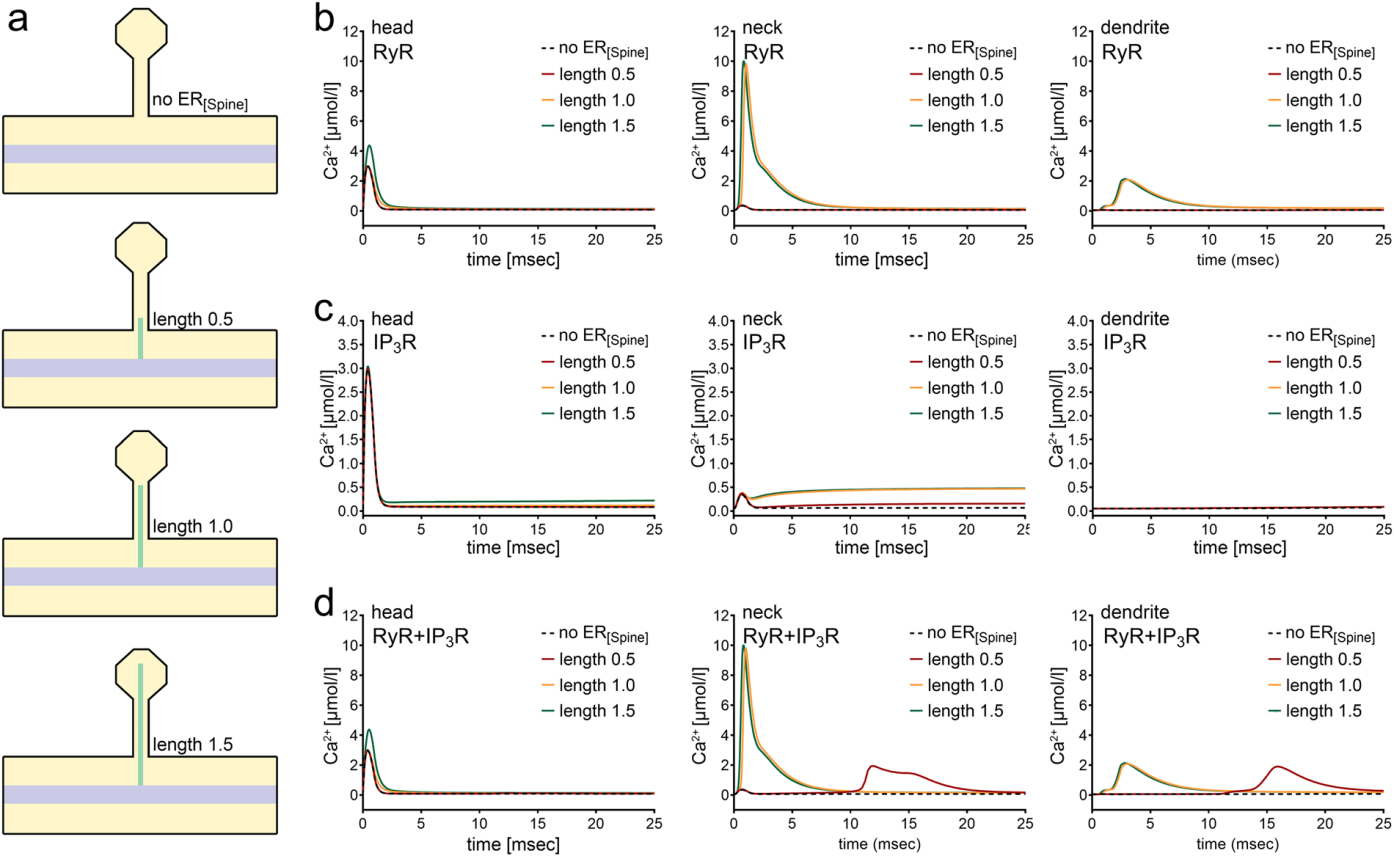
Differential effects of spine ER carrying ryanodine receptor (RyR) and inositol trisphosphate receptor (IP_3_R) on spine-to-dendrite Ca^2+^ signaling. (**a**) Schematics of spine ER morphologies used to investigate the role of RyR- and IP_3_R-mediated Ca^2+^ release. Spine ER width is kept constant, while the length is varied between 0–1.5 μm. (**b**) Ca^2+^ profiles in response to 1 ms Ca^2+^ influx into the spine head for the four configurations depicted in (a) in the spine head, neck, and dendrite (from left to right) for RyR-only spine ER. (**c**) Ca^2+^ profiles for the four configurations depicted in **(a**) in the spine head, neck, and dendrite (from left to right) for IP_3_R-only spine ER. Note that the plateau in the neck profile appears to be small compared to RyR-induced dynamics, as the plateau effect is overshadowed by RyR dynamics in (b) and (d). (**d**) Ca^2+^ profiles for the four configurations depicted in (**a**) for spine ER carrying both RyR and IP_3_R.

We next tested for the role of RyR-containing ER introduced to the spine compartment. Indeed, RyR promoted and even amplified spine-to-dendrite Ca^2+^ signals in our experimental setting (1 ms initial Ca^2+^ release illustrated in Fig. 3): For an ER length of 1.5 μm (width 0.036 μm; cf. Fig. 3a and Table 1), an approximately 20fold increase in [Ca^2+^] was measured in the spine neck (as compared to the purely passive spine ER setting), while up to twice the amount of Ca^2+^ initially released into the spine head was observed in the dendrite (Fig. 3b; see also supplemental movie). In addition, an increase in spine head [Ca^2+^] was observed as soon as the ER reached the border between the spine neck and head, while [Ca^2+^] in the neck and dendrite was comparable under these conditions. RyR-ER at the base of the spine (ER length 0.5 μm) had no apparent effect on spine-to-dendrite Ca^2+^ communication (Fig. 3b). These simulations indicate that the precise position of RyR-containing ER could have an impact on spine-to-dendrite Ca^2+^ signaling and Ca^2+^ signal amplification.

**Table 1:** Geometrical parameters of the spine model.

Parameter name	Description	Value (range) [μm]
dendrite radius	radius of dendrite segment	0.45
dendrite length	length of dendrite segment	10.0
spine neck radius	radius of spine neck	0.08
spine neck length	length of spine neck	0.7
spine head radius	radius of spine head	0.29
dendrite ER radius	radius of dendritic ER	0.11
dendrite ER length	length of dendritic ER	8.0
spine ER radius	radius of spine neck ER	0.036–0.054
spine ER length	length of spine neck ER	0–1.7
spine ER head radius	radius of spine ER head	0–0.12

All length parameters are given in units of μm.

### IP_3_ receptors introduce protracted Ca^2+^ waves in the spine head and neck

While it has been argued that spine ER may not contain IP_3_Rs in hippocampal neurons^10^, our computational approach enabled us to evaluate the behavior of IP_3_R-only spine ER. Fig. 3c shows a slow rise in [Ca^2+^] in the head and neck of spines in response to 1 ms Ca^2+^ influx and simultaneous onset of a 200 ms IP_3_ release, which became more prominent as the ER reached the spine head. In our simulations, IP_3_R-only spine ER, similar to a passive ER, does not have the ability to initiate strong spine-to-dendrite communication. Interestingly, although much weaker as compared to RyR-containing ER (cf. Fig. 3b), a small Ca^2+^ increase was even observed with ER of length 0.5 μm. We attribute this difference between RyR- and IP_3_R-containing ER to (1) the presence of Ca^2+^ buffers, which scavenge Ca^2+^ and limit its reach, while IP_3_ has a slower decay rate compared to Ca^2+^, and therefore a longer reach toward the dendrite, and (2) the fact that IP_3_R is activated at lower [Ca^2+^] in the presence of IP_3_. Thus, IP_3_R-mediated Ca^2+^ signals are weaker than RyR-Ca^2+^ signals, but are sensitive to low [Ca^2+^] even at spine ER positions distant from the synapse.

### Combining RyR and IP_3_R can cause delayed Ca^2+^ signal reverberation

Based on the results above, we speculated that the slow protracted IP_3_R-mediated Ca^2+^ -response could trigger RyR-mediated Ca^2+^ release from the ER in situations where RyR-only ER is not sufficient to promote spine-to-synapse communication. Thus, IP_3_R-mediated Ca^2+^ responses could support RyR-mediated Ca^2+^ signaling between spines and dendrites.

To test this hypothesis, we repeated our simulations with spine ER containing both RyR and IP_3_R. ER positions in the neck and head of the spine elicited Ca^2+^ dynamics that were comparable to the RyR-only simulations (Fig. 3d). When, however, the RyR/IP_3_R-containing ER was positioned at the base of the spine, an additional protracted Ca^2+^ response was observed, which propagated back toward the spine head compartment, but dissipated along the way due to Ca^2+^ buffering. This result is in line with the literature disclosing ER-mediated IP_3_-dependent protracted Ca^2+^ signals, which may promote long-term depression of excitatory neurotransmission, e.g.^11,23,24^.

### The precise position of RyR-containing ER may affect the timing of Ca^2+^ signals

**Figure 4:**
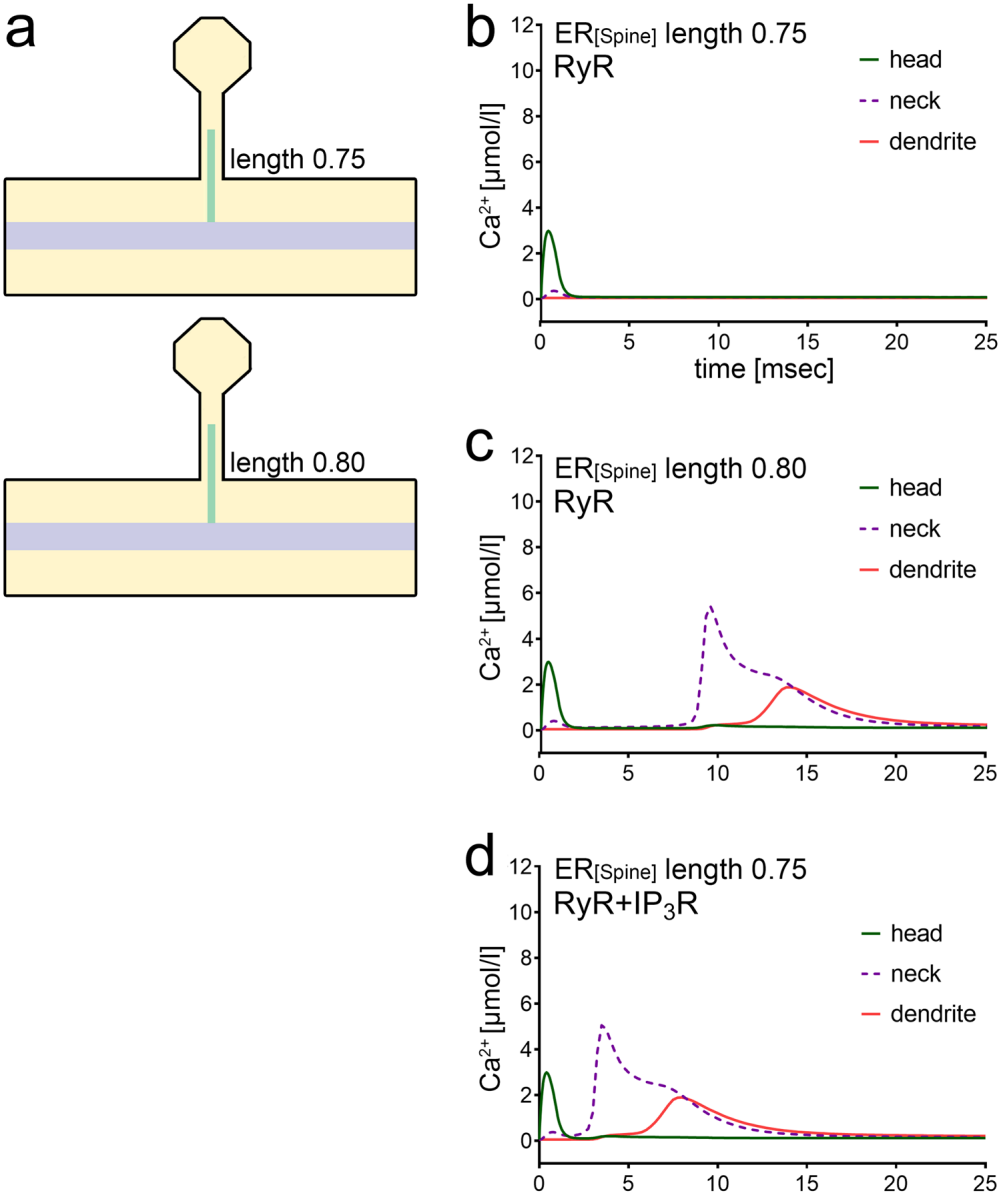
Critical spine ER transition lengths regulate all-or-nothing spine-to-dendrite communication. (**a**) Schematics of three morphological configurations designed to illustrate Ca^2+^ signals around critical ER transition lengths in response to 1 ms Ca^2+^ influx into the spine head. **(b)** For a RyR-containing ER of length 0.75 μm, no Ca^2+^ communication to the dendrite is detected. **(c)** Increasing the RyR-containing ER to a length of 0.8 μm surpasses a critical spine ER length to trigger spine-to-dendrite communication. At this critical length, [Ca^2+^] in the vicinity of the ER membrane is elevated just above the CICR threshold. This occurs after the initial dampened and dilated (by diffusion) Ca^2+^ signal, visible in the first milliseconds, propagates to the ER membrane and eventually triggers delayed CICR at roughly 8 *ms*. In the roughly 5 *ms* period leading to store release, one can observe the slow elevation of local cytosolic Ca^2+^ concentration that eventually surpasses the threshold necessary to trigger self-reinforcing Ca^2+^ release from the ER. **(d)** Adding IP_3_R to the ER allows for spine-to-dendrite signals at an ER length for which RyR-only ER is not capable of transmitting a signal to the dendrite (see panel (b) of this figure). The exact position of this transition zone depends on the initial Ca^2+^ release in the spine head (cf. Supplemental Fig. S2).

Motivated by the observation that ER positioning affects Ca^2+^ signals, we next determined the position of RyR spine ER at which spine-to-dendrite communication and Ca^2+^ signal amplification occurs. Figure 4 shows the transition that occurs for a 1 ms Ca^2+^ influx when growing the ER beyond a critical length. While no Ca^2+^ signal can be detected in the dendrite for an ER length of 0.75 μm in the RyR-only case (Fig. 4a,b), spine-to-dendrite communication is detectable for a length of 0.8 μm (Fig. 4a,c). While the exact position of this transition zone depends on the initial Ca^2+^ release in the spine head (i.e., total number of Ca^2+^ ions and release current density), the effects of the spine ER within this critical zone are robust. In case of a prolonged, i.e., 150 ms Ca^2+^ influx, the transition is found between ER lengths of 0.4 μm and 0.45 μm (c.f., Supplemental Fig. S2). Interestingly, a delay in the Ca^2+^ signal occurs at these transition positions, which can be attributed to the fact that it takes several milliseconds at this transition length for Ca^2+^ to reach the critical threshold that triggers RyR-mediated Ca^2+^ release from the ER.

Consistent with the results described above, IP_3_R- and RyR-containing ER at a position, which does not elicit RyR-only responses, triggered the described delayed IP_3_R-mediated RyR-dependent Ca^2+^ response (Fig. 4d). These findings suggest that the precise position of RyR-(IP_3_R)-containing ER (1) enables spine-to-dendrite Ca^2+^ signaling, (2) amplifies the Ca^2+^ signal, and may even (3) modulate the exact timing of the Ca^2+^ signal. Considering that the outcome of plasticity may critically depend on such timing^25–27^, i.e., coincidence detection, this appears to be a relevant observation.

### RyR-ER-dependent spine-to-dendrite Ca^2+^ coupling does not depend on the length of the spine

**Figure 5:**
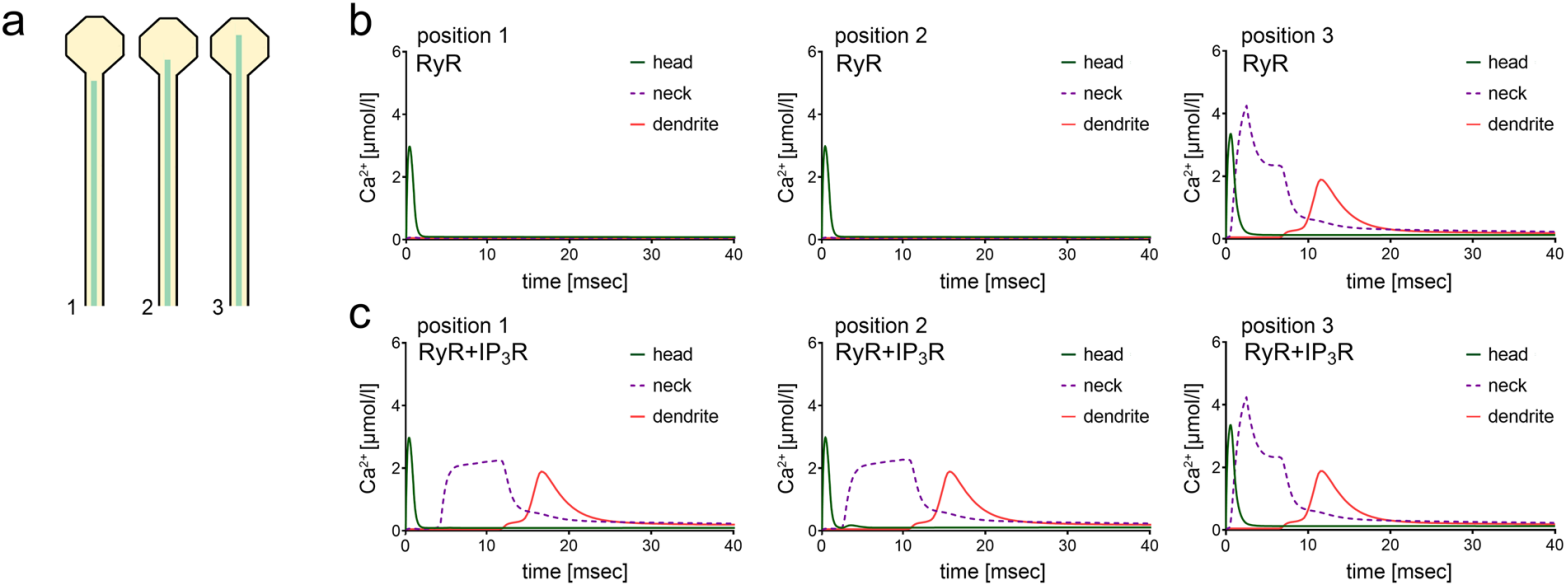
Precisely positioned active ER enables spine-to-dendrite Ca^2+^ communication in a very long spine. (**a**) Illustration of the three ER positions used for the simulations in (b) and (c). A very long spine neck (10 μm) was employed to investigate whether neck length is a limiting factor for ER-mediated spine-to-dendrite Ca^2+^ communication. **(b)** Simulations for a RyR-only ER at positions 1, 2 and 3 in response to 1 ms Ca^2+^ influx into the spine head (illustrated in panel (a) of this figure). **(c)** Addition of IP_3_R enables spine-to-dendrite Ca^2+^ communication at ER positions farther away from the synapse. Note the comparable Ca^2+^ profiles at position 3 between RyR-only and RyR+ IP_3_R ER.

To test for the role of spine length, we carried out a series of simulations in which a very long spine, i.e., 10 μm spine neck length, was used. All other spine parameters were kept constant. The following major conclusions were drawn from this series of simulations: (1) RyR-ER couples and amplifies Ca^2+^ signals in dendrites and very long spines, (2) RyR-dependent spine-to-dendrite Ca^2+^ signal coupling occurs once the ER reaches far enough into the spine (Fig. 5b, position 3), (3) spine ER reaching even further into the spine makes spine head Ca^2+^ levels increase, (4) at a position that does not show RyR-dependent Ca^2+^ release from intracellular stores, introducing IP_3_R establishes a slightly delayed spine-to-dendrite communication (Fig. 5b,c, positions 1, 2). (5) Positioning RyR-only ER at the critical transition length – depending on the initial Ca^2+^ release in the spine head – has a similar “delaying” effect on Ca^2+^ signals (cf. Fig. 4).

### Role of spine RyR-ER in spine head Ca^2+^ homeostasis during plasticity

**Figure 6:**
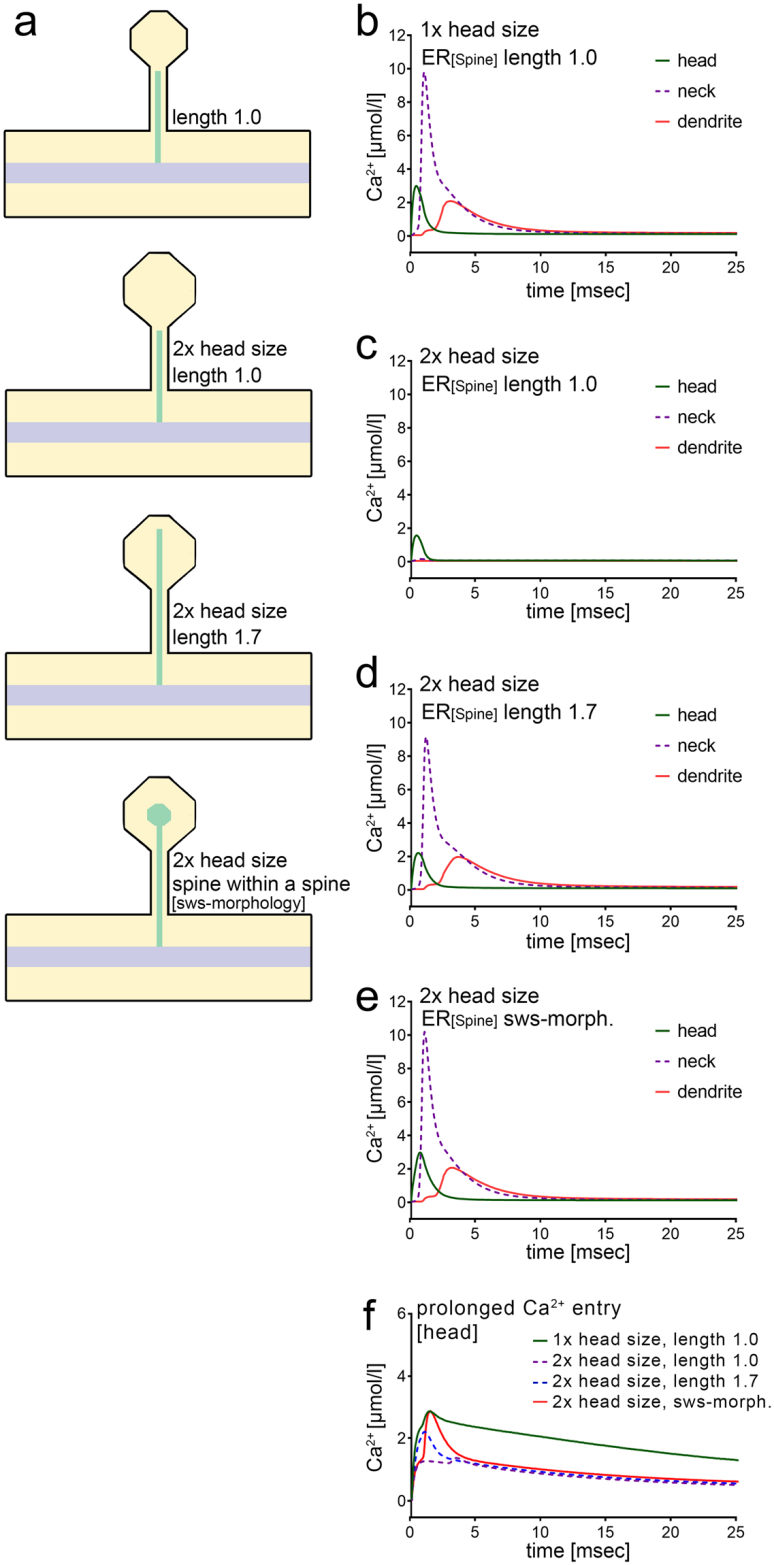
RyR-containing ER compensates increased spine head size. (**a**) Illustration of the ER morphologies used for the simulations in (**b**–**e**). (**b**,**c**) Increasing the spine head volume by a factor of 2 disables spine-to-dendrite Ca^2+^ communication in response to a 1 ms Ca^2+^ influx into the spine head. The peak [Ca^2+^] in the spine head is significantly reduced. **(d)** Increasing the ER length restores spine-to-dendrite communication. However, the original peak [Ca^2+^] in the head, neck, and dendrite cannot be restored to the original profiles in (b). **(e)** Introducing a widened terminal segment of the spine ER (“spine-within-spine” morphology) restores the original Ca^2+^ profiles. (**f**) For simulations with 10 ms Ca^2+^ release, the peak amplitude of the Ca^2+^ signal in the spine head is also restored with the “spine-within-spine” ER morphology. The decay dynamics in the head, however, become sharper in comparison, due to depletion of the intracellular calcium store.

Finally, we tested for the effects of an increase in spine head volume, as seen after the induction of synaptic plasticity (e.g.^28–30^). Considering unchanged Ca^2+^ entry, we wondered whether changes in ER morphology, i.e., ER position and size, compensate for changes in spine-to-dendrite Ca^2+^ signaling as the size of the spine head increases. As illustrated in the spine schematics (Fig. 6a), spine head volume was increased by a factor of 2. Depending on the position of the spine ER, this can lead to a loss of spine-to-dendrite communication as well as a considerable decrease in [Ca^2+^] in the spine head (Fig. 6b,c).

Increasing the length of the ER (length 1.1 μm; width 0.036 μm) or changing the width of the ER (length 1.0 μm; width 0.054 μm) reactivated spine-to-dendrite communication upon an initial 1 ms Ca^2+^ release in the spine head (Fig. 6d). However, it is not possible to fully restore the original Ca^2+^ profile, even when the ER is grown all the way close to the Ca^2+^ entry site (length 1.7 μM, width 0.036 μM; Fig. 6d).

Based on systematic evaluation, we finally determined that a selective volume increase in the ER segment located in the spine head (“spine-within-spine” morphology) leads to the best possible recovery of the Ca^2+^ profile in the spine head, neck and dendrite under conditions of increased spine head volume (Fig. 6e). For simulations with Ca^2+^ release of longer duration, the peak amplitude of the Ca^2+^ signal in the spine head is also restored with the “spine-within-spine” ER morphology. The decay dynamics in the head, however, become sharper in comparison (Fig. 6f). The reason lies in the limited ER Ca^2+^ store capacity, which rapidly depletes in our experimental setting. In the tested scenarios, dendritic dynamics were restored by morphological reorganization of the spine ER, which did not require a “spine-within-spine” morphology. Hence, complex changes in spine ER morphology seem to be required to restore Ca^2+^ homeostasis in the spine head, while precise positioning of the ER suffices to restore spine-to-dendrite Ca^2+^ communication.

## Discussion

The present study highlights how functional and structural spine ER properties may affect spine-to-dendrite Ca^2+^ signaling. While precise positioning of RyR-(IP_3_R)-containing spine ER has a major impact on spine-to-dendrite Ca^2+^ communication, affecting both the strength and timing of the signal, growth of the spine neck or the spine head can cause a disruption of Ca^2+^ signaling. Eventually, comparable Ca^2+^ profiles can be restored by changes in ER morphology, i.e., position and size. These restoration effects could be demonstrated for Ca^2+^ entry profiles with different strength/duration. The all-or-none transition points as shown in Figs 4 and 5 are detectable for all tested entry profiles, the transition points, however, shift closer towards or farther away from the spine head, depending on the Ca^2+^ entry profile. It appears that not the absolute length or volume of the spine neck and head, respectively, determine the nature of spine-to-dendrite Ca^2+^ communication, but rather the relationship between spine morphology and spine ER morphology (“spine-within-spine” morphology). The presence of RyR and IP_3_R is important in this context. Once critical distances between the postsynaptic density and the ER are overcome and/or volume ratios between spine head and ER in the spine head are met, the synapse regains its previous spine-to-dendrite Ca^2+^ communication.

The role of spine ER in synaptic plasticity remains a matter of debate. For example, controversial results exist with respect to the relevance of ER Ca^2+^ stores in synaptic calcium transients^11,28,31–35^. This is in part explained by technical limitations in simultaneously visualizing (1) dendritic spine morphology, (2) presence and precise position of spine ER, and (3) in releasing reproducible amounts of Ca^2+^ while (4) carrying out Ca^2+^ imaging at high temporal and spatial resolution. Also, it is currently not possible to systematically assess the relevance of individual spine and ER parameters, as they are not easy to manipulate in biologically complex systems. We used a computational approach to compare ER-negative and ER-positive spines and to assess the role of spine ER morphology in spine Ca^2+^ transients. We show that critical ER lengths can be determined for specific spine geometries, functioning as a binary switch for spine-to-dendrite signaling. While the precise position depends on a given Ca^2+^ entry profile, beyond this all-or-nothing regulation, ER positioning can also function at the more refined level of timing. Our simulations suggest that the timing of a dendritic Ca^2+^ signal is determined by ER position and RyR/IP_3_R configuration. In some cases, even signals reverberating in the spine neck can be detected. In addition, peak amplitudes of Ca^2+^ transients are affected. Considering the relevance of Ca^2+^ signaling in synaptic plasticity and coincidence detection, i.e., spike-timing-dependent plasticity^25–27^, our findings imply that precise positioning of the ER could influence the duration, strength and direction of plasticity^36–38^. Naturally, further work (including improvement/development of new experimental techniques) is required to address this interesting hypothesis.

Another intriguing finding of our study concerns the role of spine ER in spine head Ca^2+^ homeostasis. We provide initial evidence that ER morphologies compensate for changes in spine morphology. This set of simulations also indicates that changes in ER morphology/volume in the area between the spine neck and spine head most effectively modulate Ca^2+^ signaling. Interestingly, the peak amplitude of the Ca^2+^ signal in the spine head but not the decay dynamics can be restored, specifically in the case of prolonged Ca^2+^ influx durations. This observation is attributed to a limited Ca^2+^ storage capacity, i.e., a rapid depletion of the intracellular store in our simulations, which “sharpens” the Ca^2+^ kinetics in the spine head. Whether this observation is relevant for ER-containing spines that undergo plasticity needs to be determined. It is also possible that additional molecular mechanisms may account for the limited Ca^2+^ capacity in order to maintain homeostasis, e.g., changes in SERCA and/or SOCE.

It is worth noting in this context that spine ER can assume peculiar morphological conformations that may resemble the herein described “spine-within-spine” ER morphology. The spine apparatus organelle is found in a subset of dendritic spines, consisting of stacked ER, which is typically located in the spine neck and head^39–42^. While its role in local protein synthesis is still debated, a link has been established between the spine apparatus and intracellular Ca^2+^ stores^9,28,43–45^. Indeed, using the actin-binding protein synaptopodin, which is a marker and essential component of the spine apparatus^46,47^, evidence has been provided that synaptopodin-associated Ca^2+^ transients from intracellular stores modulate the ability of neurons to express synaptic plasticity^28,48–50^. Similar to spine ER, the spine apparatus appears to be a dynamic structure that leaves and enters individual spines^28^ and changes its size, i.e., stack number^51^. Moreover, evidence has been provided that the spine apparatus is part of a Ca^2+^-dependent negative-feedback mechanism mediating homeostatic synaptic plasticity^51^ and that the size of spine apparatuses can change under pathological conditions such as systemic inflammation^52^ or experimentally induced seizures^53^. Apparently, our current findings motivate a rigorous study of the importance of stacked, membrane-infolded ER architectures (potentially to increase a surface to volume ratio). We are confident that these future studies will shed new and important light on the relevance of ER conformation in Ca^2+^ wave segregation and propagation and may thus provide new insight into the functional significance of ER derivatives/specializations, such as spine apparatuses^39,41,42^, cisternal organelles^54–60^, or subsurface organelles in dendrites and cell bodies^61–63^.

While we addressed basic principles of spine ER reorganization using simplified morphologies in this study, it will now be important to also employ more complex, i.e., realistic spine and ER morphologies based on super-resolution microscopy and/or serial electron microscopy. These models should also consider complex synaptic activity, i.e., plasticity-inducing AMPA-R-, NMDA-R- and mGluR-mediated Ca^2+^ signals. Yet, the results of the present study show that the major conclusions are robust across various Ca^2+^ release profiles. Since more complex effects may arise, e.g., from local depletion of intracellular Ca^2+^ stores and ORAI-STIM1-mediated SOCE, it will be important to also integrate these findings into models that also account for dendritic, somatic and axonal ER configurations. It is well established that space-time integration of Ca^2+^ signals originating at multiple spines plays an important role in Ca^2+^ signaling toward the soma and nucleus^64,65^. Hence, it is conceivable that the precise nature of the timing and the waveform of synaptically induced Ca^2+^ signals are relevant not only for spine-to-dendrite communication, but also for inter-synaptic and synapse-to-nucleus communication. Thus, spine ER positioning along entire dendritic branches and within multiple synaptic spines must be considered. The nature of structure/function interplay demands an inclusion of the three-dimensional intracellular architecture in order to capture the ways in which cellular organization can influence biochemical (and potentially electrical^66^) signals. The parametric geometry design approach developed for this study was included in the simulation toolbox NeuroBox^20^. This modular framework could be extended for future studies on more complex surface/volume/distance law models that integrate and test for the relevance of complex activity patterns on spine, dendrite and somatic ER morphologies in Ca^2+^ homeostasis and synapse-to-nucleus communication.

## Methods

All necessary components were implemented in the simulation toolbox NeuroBox^20^.

### NeuroBox spine generator

NeuroBox is a simulation toolbox that combines models of electrical and biochemical signaling on one- to three-dimensional computational domains. NeuroBox allows the definition of model equations, typically formulated as ordinary and partial differential equations, of the cellular computational domain and specification of the mathematical discretization methods and solvers^67^. Built with VRL-Studio^68^, NeuroBox offers user interface workflow canvases to control the simulation workflow and all biological and numerical parameters.

A novel spine generator using a parametric design approach was developed and implemented in NeuroBox, that allowed us to systematically vary the morphology of a spine (as well as the endoplasmic reticulum) and study its influence on the intracellular Ca^2+^ dynamics (see Sec. “model equations”–“membrane transport mechanisms”). The resulting partial differential equations with membrane mechanisms on the endoplasmic and plasma membrane were solved using a finite volume discretization and a parallel iterative solver (see Sec. “numerical methods”).

In the numerical simulation framework UG 4^67^, a computational domain ${\rm{\Omega }}\in {{\mathbb{R}}}^{n}$, *n* ∈ {1, 2, 3}, is represented by a piecewise linear approximation Ω_*h*_ (“grid”). All grid-related data structures and algorithms are implemented in the UG 4 core library *lib*_ *grid* which also constitutes the basis for the UG 4 plugin and cross-platform meshing software ProMesh^69,70^. *lib*_ *grid* features state-of-the-art grid generation data structures and algorithms which were incorporated in a consecutive workflow to automatically construct 3D grid variations of a dendritic segment including the ER and spine with corresponding spine ER. To this end, the spine generator utilizes the *lib*_ *grid* functionality for basic geometric element initialization and manipulation, i.e., insertion/deletion of vertices, edges, faces and volumes, as well as translation and scaling. Furthermore, composite functions for creating and extruding simple geometric objects like circles, as well as sophisticated grid generation algorithms for constrained Delaunay tetrahedrization^71,72^ are accessed in the ProMesh plugin. A Delaunay tetrahedrization ${\mathscr{S}}=\{{T}_{1},\mathrm{...},{T}_{M}\}$ is a special kind of tetrahedrization in which every tetrahedron *T*_*i*_ complies with the *Delaunay condition*, i.e., the unique circumsphere of each *T*_*i*_, which passes through the four tetrahedral vertices, does not contain any vertices of the grid in its interior (Fig. 1d). This leads to high-quality grids which avoid tetrahedra with particularly acute or obtuse interior angles^73^, an essential grid property for accurate approximation and fast solution in numerical simulation^74–76^.

The user can specify 10 characteristic geometric parameters to specify the individual morphology of the spine grid output (Table 1). The generated grids are written to the native UG 4 file format UGX and can be viewed and modified using the GUI version of ProMesh. The fundamental workflow can be summarized as follows:

1. The exterior dendrite and interior ER structures are constructed around the origin (0.0, 0.0, 0.0) using the ProMesh create-circle approximation with a chosen default resolution of 8 rim vertices and user-specified radii (Fig. 1e).
2. The dendritic and ER circles, respectively, are then successively extruded along the z-axis while at the same time creating quadrilateral faces enclosing the emerging cylinder barrels using user-specified lengths (Fig. 1f).
3. The previous process is interrupted by an in-between extrusion step for creating a measurement zone of user-specified spine neck length around the spine ER placed at the user-specified z-coordinate for the spine position.
4. At the z-coordinate for the spine position, spine ER and neck are generated by a circular remeshing of the local ER and dendritic surface grid, and subsequent extrusion along the y-axis, creating quadrilateral faces enclosing the emerging cylinder barrels using specified lengths.
5. The spine head is placed on top of the neck by continued extrusion along the y-axis. Subsequently, the cylindrical spine head vertices are projected to spherical coordinates around the head barycenter using the head radius.
6. The remaining planar holes at the dendrite and ER cylinder top and bottom are triangulated to close the encapsulated surface geometry (Fig. 1g).
7. Surface elements are selected automatically by their coordinates in order to be assigned to individual subsets for access during numerical simulation.
8. Given the piecewise linear closed and encapsulated surface geometry, the corresponding volume grid is generated using constrained Delaunay tetrahedrization allowing for an individual subset assignment of tetrahedral elements which are separated by lower dimensional subsets.

### Model equations

Three-dimensional spatio-temporal Ca^2+^ and inositol trisphosphate (IP_3_) dynamics in the intracellular space are modeled by a system of diffusion-reaction equations described in the following. The boundary conditions for this partial differential equation system are specified by Ca^2+^ - and IP_3_-dependent flux boundary conditions described in Sec. “Membrane transport mechanisms”.

The model considers the quantities *calcium* (cytosolic (*c*_*c*_) and endoplasmic (*c*_*e*_)), *calbindin-D*_*28k*_ (*b*), and IP_3_ (*p*), which is required to model IP_3_ receptors embedded in the endoplasmic membrane. Mobility in the cytosol/ER is described by the diffusion equation

1 $$\frac{\partial u}{\partial t}=\nabla \cdot (D\nabla u)$$

where *u*(*x*, *t*) stands for the the four quantities mentioned above. The diffusion constants *D* are defined using data from^77,78^.

The interaction between cytosolic Ca^2+^ and calbindin-D_28k_ (CalB) is described by

2 $${{\rm{Ca}}}^{2+}+{\rm{CalB}}\underset{{\kappa }_{b}^{-}}{\overset{{\kappa }_{b}^{+}}{\rightleftharpoons }}[{{\rm{CalBCa}}}^{2+}].$$

The rate constants ${\kappa }_{b}^{-}$ and ${\kappa }_{b}^{+}$ are given in Table 2. While CalB has four distinct high-affinity Ca^2+^-binding sites^79^, we currently treat it as though it had only one, at the same time quadrupling its concentration in our model. This amounts to assuming that all four binding sites are essentially equal and binding is non-cooperative (though data by^80,81^ indicate this might not neccessarily be the case).

**Table 2:** Model parameters and initial values.

Initial and equilibrium values			SERCA pumps		
*c*_*c*_	50 nM	(chosen)	*I*_*S*_	6.5 × 10^−21^ molμMs^−1^	^99^ (adapt.)
*c*_*e*_	250 μM	(chosen)	*K*_*S*_	180 nM	^86^
*c*_*o*_	2 mM	(chosen)	*ρ*_*S*_	2390 μm^−2^	^98^ (approx.)
*p*	40 nM	^95^	**PMCA pumps**		
*b* ^tot^	40 μM	^96^	*I*_*P*_	1.7 × 10^−23^ mol s^−1^	^87^
**Diffusion**/**reaction**			*K*_*P*_	60 nM	^100^
*D*_*c*_	220 μm^2^ s^−1^	^77^	*ρ*_*P*_	500 μm^−2^	(estim.)
*D*_*p*_	280 μm^2^ s^−1^	^77^	**NCX pumps**		
*D*_*b*_	20 μm^2^ s^−1^	^78^	*I*_*N*_	2.5 × 10^−21^ mol s^−1^	^87^ (adapt.)
${\kappa }_{b}^{-}$	19 s^−1^	^96^	*K*_*N*_	1.8 μM	^87^
${\kappa }_{b}^{+}$	27 μM^−1^ s^−1^	^96^	*ρ*_*Ν*_	15 μm^−2^	(estim.)
*κ*_*p*_	0.11 s^−1^	^97^			
*p* ^*r*^	40 nM	^95^	**leakage**		
**IP**_**3**_ **R channel**			*v*_*l*, *e*_	38 nms^−1^	(calc.)
*d*_1_	0.13 μM	^83^	*v*_*l*, *p*_	4.5 nms^−1^	(calc.)
*d*_2_	1.05 μM	^83^	**calcium/IP**_**3**_ **release**		
*d*_3_	0.94 μM	^83^	${j}_{c}^{{\rm{rls}}}$	1.0 × 10^−16^ mol s^−1^ μm^−2^	1 ms release
*d*_5_	82.3 nM	^83^		5.0 × 10^−17^ mol s^−1^ μm^−2^	10 ms release
*ρ*_*Ι*_	17.3 μm^−2^	^98^	*τ*_rls_	10 ms	
${I}_{I}^{{\rm{ref}}}$	1.1 × 10^−19^ mol s^−1^	^82^ (extrapol.)	*τ*_NMDAR_	150 ms	
**RyR channel**			*p*_NMDAR_	8.15 × 10^−3^ μm^3^ s^−1^	
${k}_{a}^{-}$	28.8 s^−1^	^84^	$\tilde{V}$	13.4 mV	
${k}_{a}^{+}$	1500 μM^−4^ s^−1^	^84^	${j}_{p}^{{\rm{rls}}}$	5.0 × 10^−18^ mol s^−1^ μm^−2^	^92^ (adapt.)
${k}_{b}^{-}$	385.9 s^−1^	^84^			
${k}_{b}^{+}$	1500 μM^−3^ s^−1^	^84^			
${k}_{c}^{-}$	0.1 s^−1^	^84^			
${k}_{c}^{+}$	1.75 s^−1^	^84^			
*ρ*_*R*_	3.0 μm^−2^	(estim.)			
${I}_{R}^{{\rm{ref}}}$	3.5 × 10^−18^ mol s^−1^	^85^ (approx.)			

The equations for cytosolic Ca^2+^ and CalB are thus given by

3 $$\frac{\partial {c}_{c}}{\partial t}={D}_{c}{\rm{\Delta }}{c}_{c}\,+\,({\kappa }_{b}^{-}({b}^{{\rm{tot}}}-b)-{\kappa }_{b}^{+}\,b\,{c}_{c}),$$

4 $$\frac{\partial b}{\partial t}={D}_{b}{\rm{\Delta }}b\,+\,({\kappa }_{b}^{-}({b}^{{\rm{tot}}}-b)-{\kappa }_{b}^{+}\,b\,{c}_{c})$$

in the cytosolic domain, where the concentration of the CalB-Ca^2+^ compound is expressed by the difference of the total concentration of CalB present in the cytosol (*b*^tot^) and free CalB, the former of which is assumed to be constant in space and time (this amounts to the assumption that free and Ca^2+^ -binding CalB have the same diffusive properties). All parameters are listed in Table 2.

Exponential IP_3_ decay towards a basal IP_3_ concentration *p*^*r*^ in the cytosolic space is modeled by a reaction term that is added to the IP_3_ diffusion equation, leading to the diffusion-reaction equation

5 $$\frac{\partial p}{\partial t}={D}_{p}{\rm{\Delta }}p\,-\,{\kappa }_{p}(p-{p}^{r})$$

for IP_3_ in the cytosolic domain. Endoplasmic Ca^2+^ dynamics are modeled by simple diffusion

6 $$\frac{\partial {c}_{e}}{\partial t}={D}_{c}{\rm{\Delta }}{c}_{e}$$

in the endoplasmic domain.

### Membrane transport mechanisms

In order to study the influence of intracellular organization on Ca^2+^ signals, we include Ca^2+^ exchange mechanisms on the endoplasmic membrane (ERM) and the plasma membrane (PM). IP_3_ receptors (IP_3_R), ryanodine receptors (RyR), sarco/endoplasmic reticulum Ca^2+^ -ATPase pumps (SERCA) as well as a leakage term are modeled to describe the bi-directional exchange of Ca^2+^ across the ER membrane. For the plasma membrane we consider plasma membrane Ca^2+^ -ATPase pumps (PMCA), Na^+^/Ca^2+^ exchangers (NCX) and a leakage term. This amounts to the flux equations

7 $${j}_{{\rm{ERM}}}={j}_{I}+{j}_{R}-{j}_{S}+{j}_{l,e},$$

8 $${j}_{{\rm{PM}}}=-\,{j}_{P}-{j}_{N}+{j}_{l,p}.$$

where *j*_*I*_ is the IP_3_R flux density, *j*_*R*_ the RyR flux density, *j*_*S*_ the SERCA flux density and *j*_*l*,*e*_ the leakage flux density on the ERM, and *j*_*P*_, *j*_*N*_ and *j*_*l*,*p*_ the flux densities of PMCA, NCX, and leakage flux density of the PM, respectively. Homogeneous distributions of all exchange mechanisms were assumed, as experimental data on precise numbers and spatio-temporal distribution of these receptors within individual spines are not available.

#### IP_3_R channels

The flux density *j*_*I*_ (number of ions per membrane area and time) through the ER membrane is calculated by

9 $${j}_{I}={\rho }_{I}\cdot {p}_{I}^{o}\cdot {I}_{I},$$

where *ρ*_*I*_ is the density of IP_3_ receptors in the ER membrane, ${p}_{I}^{o}$ is the open state probability of a single channel, and *I*_*I*_ the single channel Ca^2+^ current.

The single channel current model is based on^82^, where experimental data are fitted by a Michaelis-Menten equation, and is quasi-linear in the physiologically relevant range for luminal Ca^2+^ concentrations (and below). Thus, we chose

10 $${I}_{I}={I}_{I}^{{\rm{ref}}}\frac{{c}_{e}-{c}_{c}}{{c}_{e}^{{\rm{ref}}}}$$

with a reference concentration ${c}_{e}^{{\rm{ref}}}$ well inside the admissible range.

For the open state probability, we used the model from^83^:

11 $${p}_{I}^{o}={(\frac{{d}_{2}{c}_{c}p}{({c}_{c}p+{d}_{2}p+{d}_{3}{c}_{c}+{d}_{1}{d}_{2})({c}_{c}+{d}_{5})})}^{3}$$

with kinetic parameters *d*_1_, *d*_2_, *d*_3_ and *d*_5_ (see Table 2).

#### RyR channels

Similar to the IP_3_R channels, the Ca^2+^ flux density generated by ryanodine receptor channels at the ER membrane is given by an expression of the form

12 $${j}_{R}={\rho }_{R}\cdot {p}_{R}^{o}\cdot {I}_{R},$$

where *ρ*_*R*_ is the density of RyR in the ER membrane, ${p}_{R}^{o}$ is the open state probability of a single channel, and *I*_*R*_ the single channel Ca^2+^ current.

Using the approach from^84^, we describe the single channel ionic current by

13 $${I}_{R}={I}_{R}^{{\rm{ref}}}\frac{{c}_{e}-{c}_{c}}{{c}_{e}^{{\rm{ref}}}},$$

where the reference current ${I}_{R}^{{\rm{ref}}}$ is approximated from data presented in^85^.

The open probability for RyR channels is taken from^84^ and can be calculated as the sum of the two open states *o*_1_ and *o*_2_ in the system of ordinary differential equations

14 $${o}_{1}=1-{c}_{1}-{o}_{2}-{c}_{2}$$

15 $$\frac{\partial {c}_{1}}{\partial t}={k}_{a}^{-}{o}_{1}\,-\,{k}_{a}^{+}{c}_{c}^{4}{c}_{1}$$

16 $$\frac{\partial {o}_{2}}{\partial t}={k}_{b}^{+}{c}_{c}^{3}{o}_{1}\,-\,{k}_{b}^{-}{o}_{2}$$

17 $$\frac{\partial {c}_{2}}{\partial t}={k}_{c}^{+}{o}_{1}\,-\,{k}_{c}^{-}{c}_{2}$$

with the kinetic constants ${k}_{a}^{\pm }$, ${k}_{b}^{\pm }$ and ${k}_{c}^{\pm }$ (see Table 2), that can be solved independently for every point on the surface of the ER membrane.

#### SERCA pumps

The current from sarco/endoplasmic reticulum Ca^2+^ -ATPase pumps is described by a model from^86^, which was adapted for the three-dimensional case, and gives rise to the Ca^2+^ flux density

18 $${j}_{S}={\rho }_{{\rm{S}}}\cdot \frac{{I}_{S}{c}_{c}}{({K}_{S}+{c}_{c}){c}_{e}}.$$

The model reflects the dependence of the Ca^2+^ current not only on the cytosolic concentration but also on the endoplasmic saturation. Parameter specifications can be found in Table 2.

#### PMCA pump

Using the model presented by^87^, we model the plasma membrane Ca^2+^-ATPase current as a second-order Hill-equation

19 $${j}_{P}={\rho }_{P}\cdot \frac{{I}_{P}{c}_{c}^{2}}{{K}_{P}^{2}+{c}_{c}^{2}}\mathrm{.}$$

All parameters are listed in Table 2.

#### NCX pump

For the Na^+^/Ca^2+^ exchanger current, we assume a constant Na^+^concentration at the plasma membrane, following the first-order Hill-equation used in^87^:

20 $${j}_{N}={\rho }_{N}\cdot \frac{{I}_{N}{c}_{c}}{{K}_{N}+{c}_{c}}.$$

All parameters are listed in Table 2.

#### Leakage

Both the ERM and the PM allow a leakage flux not accounted for by the above transport mechanisms. These leakage fluxes are calibrated to ensure zero membrane net flux in the equilibrium state for all simulated ions and agents. Leakage flux densities are modeled by

21 $${j}_{l,e}={v}_{l,e}\cdot ({c}_{e}-{c}_{c}),$$

22 $${j}_{l,p}={v}_{l,p}\cdot ({c}_{o}-{c}_{c}),$$

where *c*_*o*_ is the extracellular Ca^2+^ concentration, which is assumed to be constant throughout all simulations.

#### Calcium release and IP_3_ production

Calcium release is modeled as a Neumann boundary condition for the cytosolic Ca^2+^ concentration, i.e., a time-dependent influx density function defined at the postsynaptic membrane. The 1 ms release is modeled by a linearly decreasing Ca^2+^ pulse of 1 ms duration starting at an initial maximal specific current density ${j}_{c}^{{\rm{rls}}}$. The 10 ms calcium release profile was modeled as a decaying exponential influx

23 $${j}_{c}^{{\rm{rls}}}\,\exp (-\frac{t}{{\tau }_{{\rm{rls}}}})$$

with a decay constant *τ*_rls_ = 10 ms. The prolonged calcium release was modeled by an NMDA receptor model that defined a flux with time constant *τ*_NMDAR_ = 150 ms:

24 $${j}_{{\rm{NMDAR}}}={\rho }_{{\rm{NMDAR}}}\,{p}_{{\rm{\max }}}^{o}\,\exp (-\frac{t}{{\tau }_{{\rm{NMDAR}}}})\cdot {I}_{{\rm{NMDAR}}},$$

where *ρ*_NMDAR_ is the density of NMDARs in the postsynaptic membrane, ${p}_{{\rm{\max }}}^{o}$ is the maximal single-channel open probability, and the single-channel ionic current *I*_NMDAR_ is given by the Goldman-Hodgkin-Katz expression

25 $${I}_{{\rm{NMDAR}}}={p}_{{\rm{NMDAR}}}\cdot \frac{{V}_{m}}{\tilde{V}}\cdot \frac{{c}_{o}-{c}_{c}\,\exp (\frac{{V}_{m}}{\tilde{V}})}{1.0-\exp (\frac{{V}_{m}}{\tilde{V}})}\mathrm{.}$$

The product ${\rho }_{{\rm{NMDAR}}}\,{p}_{{\rm{\max }}}^{o}$ was calibrated such that the maximal expected number of open channels in the spine was one^88^. The permeability *p*_NMDAR_ was set to a value that results in a single-channel current in accordance with data published by Jahr and Stevens^89^. We supposed a constant membrane potential value *V*_*m*_ = −70 mV and used the fact that approximately 10% of the current through NMDARs is carried by Ca^2+^ at 2 mM extracellular Ca^2+^ concentrations^89^. We took into account the Mg^2+^ block that reduces the channel conductance by a factor of about twenty^90^ at physiological 0.7 mM extracellular Mg^2+^ concentrations^91^.

The production of IP_3_ is also modeled as an “influx” (as it is produced at the plasma membrane) decaying linearly over the course of 200 ms from a specific current density ${j}_{p}^{{\rm{rls}}}$. Since we only simulate on a portion of the dendrite at the base of a single spine, we do not capture diffusion across the dendritic boundaries of our geometry. While this is irrelevant for fast-buffered calcium, IP_3_ can diffuse farther and is eventually constrained by the geometric boundaries. Over time this would lead to unrealistically high accumulation of IP_3_. To adjust for this effect, IP_3_ production was reduced compared to^92^.

Values for all model parameters are gathered in Table 2.

### Numerical Methods

For numerical simulations, the four equations are discretized in space using a finite volumes method. Current densities, both synaptic and across the ER and plasma membranes, can be incorporated into the reaction-diffusion process very naturally and easily this way. We show how this is achieved using the cytosolic Ca^2+^ Eq. (3) as an example: It is reformulated (using the divergence theorem) to an integral version

26 $${\int }_{B}\,\frac{\partial {c}_{c}}{\partial t}\,dV={\int }_{\partial B}\,{D}_{c}{\nabla }^{T}{c}_{c}\cdot {n}_{\partial B}\,dS+{\int }_{B}\,({\kappa }_{b}^{-}({b}^{{\rm{tot}}}-b)-{\kappa }_{b}^{+}\,b\,{c}_{c})\,dV,$$

where *B* is a control volume that will be specified shortly, and *n*_∂*B*_ is the outward normal on the boundary of *B*. For control volumes located at the ER membrane, some portion of its boundary will coincide with the ER membrane. Since there is no diffusive flux density *D*_*c*_∇*c*_*c*_ across the ER membrane, we can simply substitute it by the ER flux density *j*_ERM_ as given in (7) in the boundary integral for this portion of the boundary. The same applies to the plasma membrane and the synapse area. The diffusive flux is set to zero on the rest of the cytosolic domain boundary. If we denote the cytosolic boundary by Γ, its ER/plasma membrane and synaptic parts by Γ_ERM_, Γ_PM_ and Γ_syn_, respectively, this yields the following equation:

27 $$\begin{array}{rcl}{\int }_{B}\frac{\partial {c}_{c}}{\partial t}\,dV & = & {\int }_{\partial B\backslash {\rm{\Gamma }}}{D}_{c}{\nabla }^{T}{c}_{c}\cdot {n}_{\partial B}\,dS+{\int }_{\partial B\cap {{\rm{\Gamma }}}_{{\rm{ERM}}}}{j}_{{\rm{ERM}}}^{T}\cdot {n}_{\partial B}\,dS\\  &  & +{\int }_{\partial B\cap {{\rm{\Gamma }}}_{{\rm{PM}}}}{j}_{{\rm{PM}}}^{T}\cdot {n}_{\partial B}\,dS\,+\,{\int }_{\partial B\cap {{\rm{\Gamma }}}_{{\rm{syn}}}}{j}_{{\rm{syn}}}^{T}\cdot {n}_{\partial B}\,dS\\  &  & +{\int }_{B}({\kappa }_{b}^{-}({b}^{{\rm{tot}}}-b)-{\kappa }_{b}^{+}\,b\,{c}_{c})\,dV\mathrm{.}\end{array}$$

Control volumes are constructed as a Voronoi-like dual tesselation of the original tetrahedral mesh by connecting the mid-points of edges, faces and volumes through planar facets. Equation (27) must hold for all control volumes, giving rise to one equation per control volume.

Time discretization is realized using a backwards Euler scheme, i.e., for each point in time *t*, the term $\frac{\partial {c}_{c}}{\partial t}$ in (27) is replaced by the discretized term $\frac{{c}_{c}(t)-{c}_{c}(t-\tau )}{\tau }$ and all quantities on the right-hand side are evaluated at time *t*. Here, *τ* is the time step size of the time discretization.

By limiting the function space to the space of continuous functions that are linear on all volumes of the original mesh, the integrals in Eq. (27) can be evaluated efficiently. Moreover, the solution can be represented by one degree of freedom per volume, so there is one equation for each degree of freedom. The system of equations arising from this procedure is nonlinear (due to the nonlinear reaction term and, more importantly, the highly nonlinear transport terms across the membranes) and is therefore linearized by a Newton iteration.

For the results we present here, the emerging linearized problems were solved using a Bi-CGSTAB^93^ linear solver preconditioned by an incomplete LU decomposition. Computations were facilitated by a domain decomposition parallelization approach and carried out using the UG 4 framework^67^ on the JURECA computer system at the Jülich Supercomputing Centre^94^.

## Electronic supplementary material

Supplemental Figures

Supplemental movie
