# Supplementary material for: Spine-to-Dendrite Calcium Modeling Discloses Relevance for Precise Positioning of Ryanodine Receptor-Containing Spine Endoplasmic Reticulum
Source: Sci Rep. 2018 Oct 23;8:15624. doi: 10.1038/s41598-018-33343-9 (PMC6199256; doi:10.1038/s41598-018-33343-9)
Supplement: Supplementary file 1 — Supplemental Figures [file 41598_2018_33343_MOESM1_ESM.pdf]

Supplemental material to the manuscript

Spine-to-Dendrite Calcium Modeling Discloses Relevance for Precise Positioning of Ryanodine-Receptor-Containing Spine Endoplasmic Reticulum

Markus Breit, Marcus Kessler, Martin Stepniewski, Andreas Vlachos, Gillian Queisser

Supplemental Figure S1

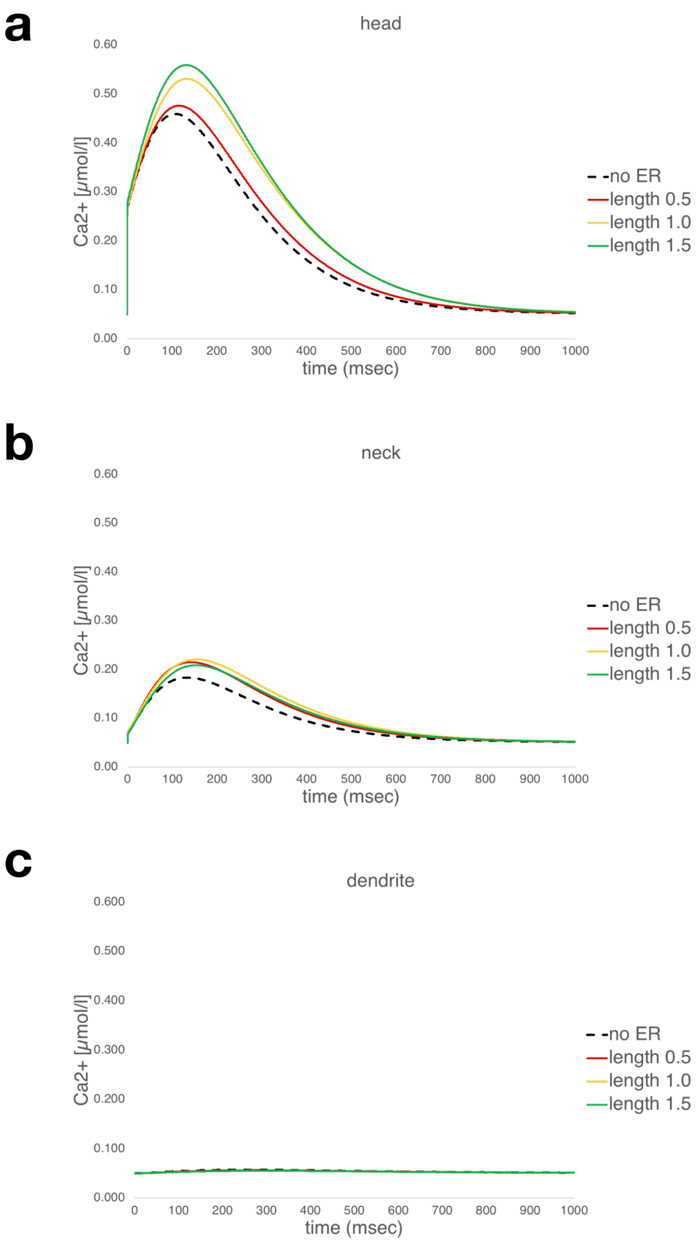

Supplemental Figure S1: Effects of passive spine ER on spine-to-dendrite Ca^{2+} signaling in response to 150 ms Ca^{2+} release into the spine head.} Comparable to the results presented in Fig. 2 and consistent with experimental data, only a small fraction of Ca^{2+} released in the head **(a)**, propagating through the neck **(b)** reaches the dendritic compartment **(c)**. When the ER is present only as a geometric obstacle, nearly identical and near-zero dendritic Ca^{2+} profiles for all ER lengths are observed.

Supplemental Figure S2

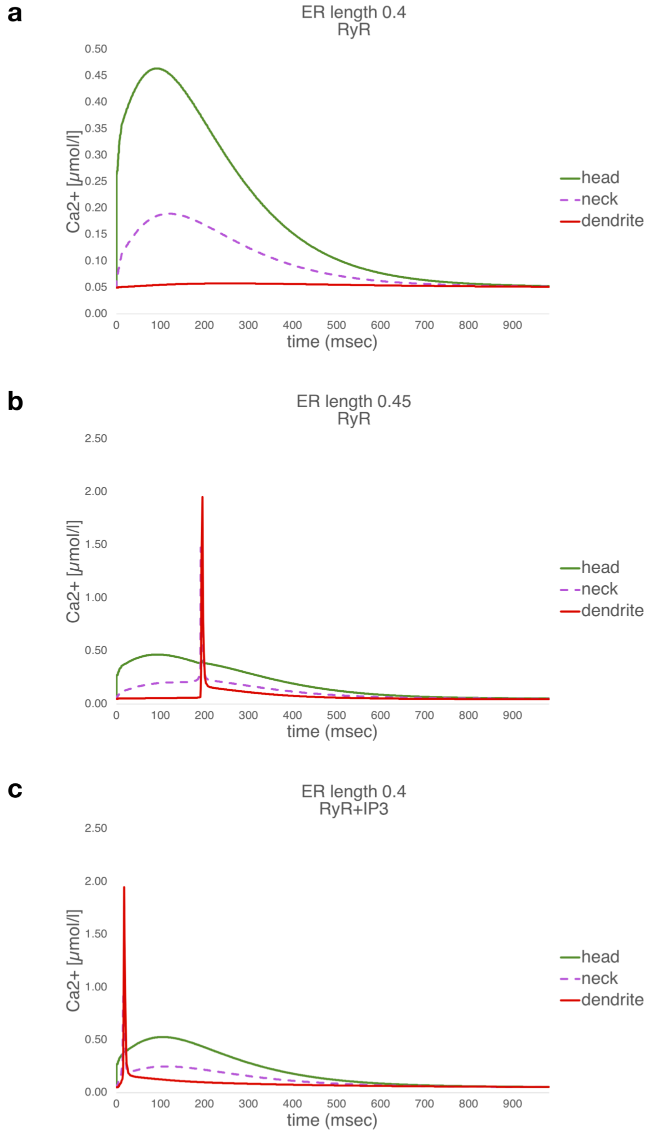

Supplemental Figure S2: Critical spine ER transition lengths regulate all-or-nothing spine-to-dendrite communication in response to 150 ms Ca^{2+} influx into the spine head. **(a)** For a RyR-containing ER of length 0.4 µm, no Ca^{2+} communication to the dendrite can be measured. **(b)** Increasing the RyR-containing ER to a length of

0.45 µm surpasses a critical spine ER length to trigger spine-to-dendrite communication. **(c)** Adding IP_3R to the ER allows for spine-to-dendrite signals at an ER length for which RyR-only ER was not capable of transmitting a signal to the dendrite (see panel (a) of this figure). While the exact position of this transition zone depends on the initial Ca^{2+} release in the spine head, the effects of the spine ER within this critical zone are robust. Note the rapid Ca^{2+} release from the ER, which ”sharpens” the signal (c.f., Figure 6f).
